# Not All Gastric Ulcers Are Malignant

**DOI:** 10.7759/cureus.26535

**Published:** 2022-07-03

**Authors:** Muhammad Sheharyar Warraich, Bashar Attar, Shazaq Khalid

**Affiliations:** 1 Medicine, John H. Stroger, Jr. Hospital of Cook County, Chicago, USA; 2 Gastroenterology and Hepatology, Rush University Medical Center, Chicago, USA; 3 Gastroenterology and Hepatology, John H. Stroger, Jr. Hospital of Cook County, Chicago, USA; 4 Medicine, Punjab Medical College, Faisalabad, PAK

**Keywords:** gastritis, vasculitis, diarrhea, gastric ulcer, eosinophilic granulomatosis with polyangiitis (egpa)

## Abstract

Eosinophilic granulomatosis with polyangiitis (EGPA) is a small- and medium-vessel vasculitis that majorly involves the respiratory tract but can potentially involve any organ system of the body. Involvement of the gastrointestinal (GI) tract can present on a spectrum. We present an interesting case of a 44-year-old man with a history of asthma and sinusitis who presented with non-specific GI symptoms and weight loss. The patient got diagnosed with EGPA. Endoscopic workup of the GI complaints revealed gastric ulcer and erosions of the upper GI tract. Biopsies of the lesions demonstrated eosinophilic infiltration suggestive of EGPA. This report represents a rare case of GI involvement of EGPA that could be histologically confirmed.

## Introduction

Eosinophilic granulomatosis with polyangiitis (EGPA), formerly known as Churg-Strauss syndrome, is a multisystem disorder characterized by chronic rhinosinusitis, asthma, and peripheral eosinophilia. Although the respiratory system is the most commonly involved organ system, EGPA can involve any organ system including the cardiovascular, renal, and gastrointestinal (GI). Cardiac involvement is typically serious and is also the leading cause of death in patients with EGPA. EGPA rarely affects children and individuals older than 65 years. This disease does not exhibit a gender predominance. The pathogenesis of EGPA is not completely understood, but it is proposed to be caused by the dysfunction of the immune system at many levels. Genetic factors are also thought to play a role in the disease process. The characteristic pathology seen with EGPA is small- and medium-vessel necrotizing vasculitis and granulomatous inflammation. There is a variety of pathologic findings seen in various organs, the most common being asthmatic bronchitis and vasculitis in the lungs, crescentic glomerulonephritis and interstitial nephritis in the kidneys, and endomyocarditis in the heart. When the GI system is involved, symptoms like abdominal pain and diarrhea are common; however, there are only a few reports with confirmed histologic involvement. We present a case of EGPA with gastric ulcer and erosions.

## Case presentation

A 43-year-old man with a history of rhino-sinusitis and adult-onset asthma presented to the emergency for bilateral lower-extremity swelling and numbness, purpuric rash in the ankle area, chronic watery diarrhea, and weight loss of two-month duration. The patient did not report the use of any non-steroidal anti-inflammatory drugs or steroids over the past couple of years. On presentation, vital signs were all within normal limits. The physical examination was significant for a non-blanchable purpuric rash on bilateral ankles with loss of sensation to light and coarse touch in the same area. Chemistry was notable for marginally low serum albumin at 3.7 g/dL and blood count differential was significant for absolute eosinophilia (2744/mL, 28%). Gastroenterology, Dermatology, and Rheumatology were consulted due to concern for an underlying vasculitic syndrome involving multiple organ systems. Dermatology obtained punch biopsies from the right lower extremity and right waistline, both of which showed skin with superficial dermal perivascular, intravascular, and interstitial eosinophilic infiltrate and mild papillary edema consistent with eosinophilic vasculitic changes.

Erythrocyte sedimentation rate and C-reactive protein were elevated at 55 mm/hour and 1.5 mg/dL, respectively. Urinalysis was unremarkable. Stool studies including *Clostridium difficile* and ova and parasites, hepatitis panel, and celiac/HIV/syphilis serologies were negative. Anti-nuclear antibody (ANA) and anti-neutrophil cytoplasmic antibodies (c-ANCA/PR3 and p-ANCA/MPO) were also negative. Angiotensin-converting enzyme level, complements, TB Quantiferon, vitamin B12, and folate levels were all within normal limits. Electromyography was performed for the sensory loss which showed sensorimotor axonal polyneuropathy.

**Figure 1:**
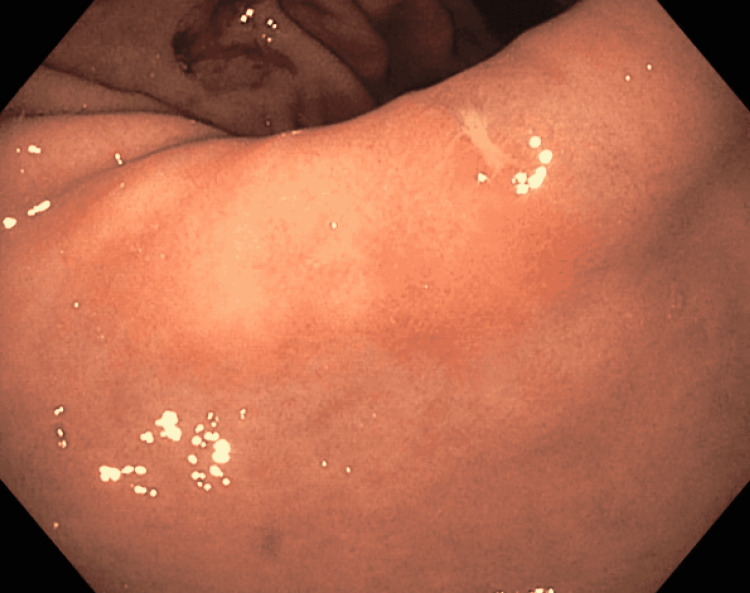
Gastric incisura ulcer

**Figure 2:**
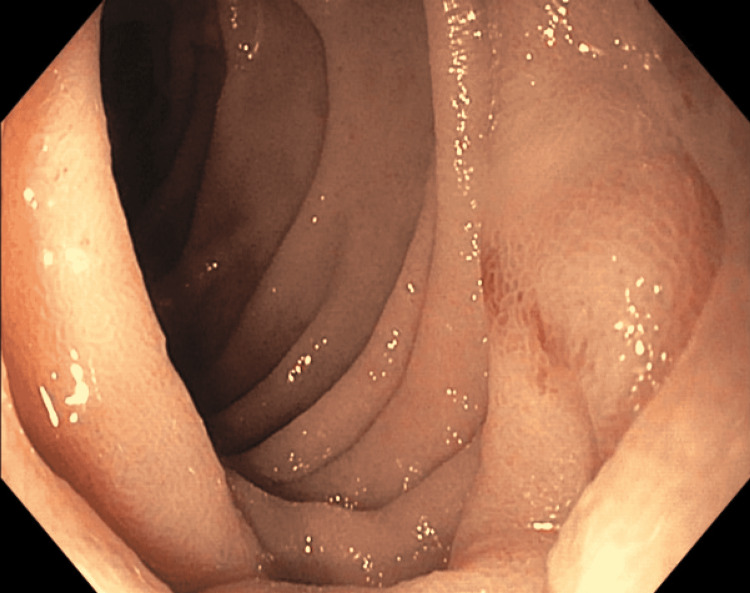
Duodenal erosion

The patient underwent esophagogastroduodenoscopy (EGD) and colonoscopy while inpatient. Colon was grossly unremarkable. EGD showed whitish plaques in the distal esophagus, gastric body and antral mucosal erythema, a clean-based ulcer at gastric incisura (Figure 1), and non-erosive duodenitis extending from the bulb to the second portion of the duodenum (Figure 2).

**Figure 3:**
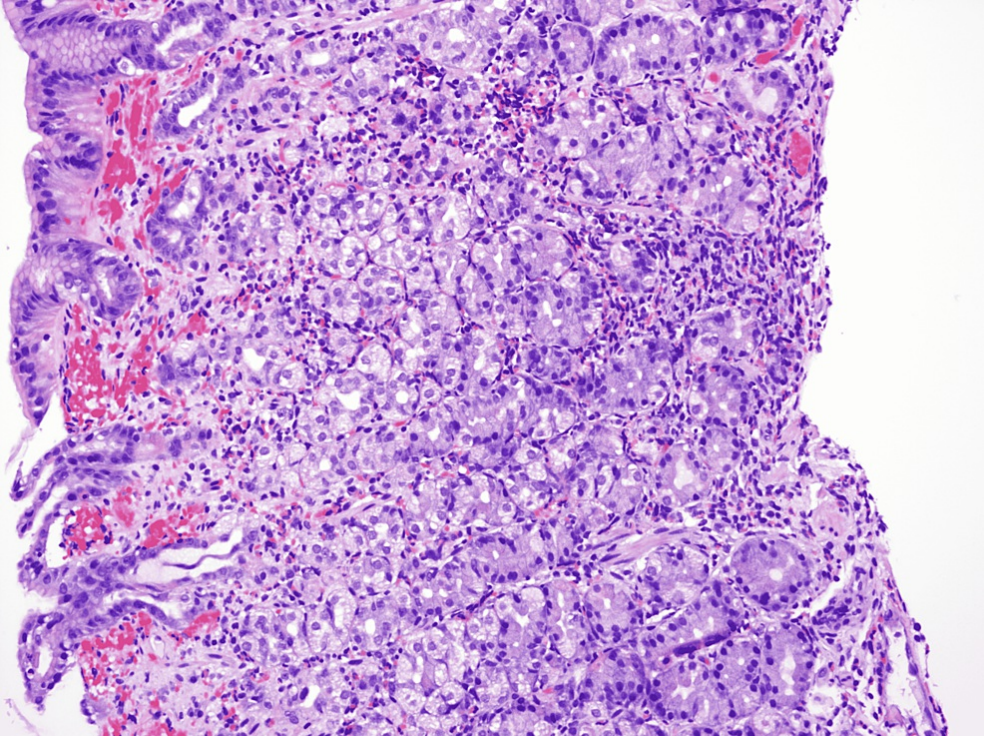
Biopsied gastric body/ulcer sample: low-magnification (200x) view Fragments of gastric body mucosa with moderate chronic gastritis containing numerous eosinophils (>60/high-power field). The eosinophilic infiltrate is arranged in a single layer and small clusters associated with a small focus of superficial erosion.

**Figure 4:**
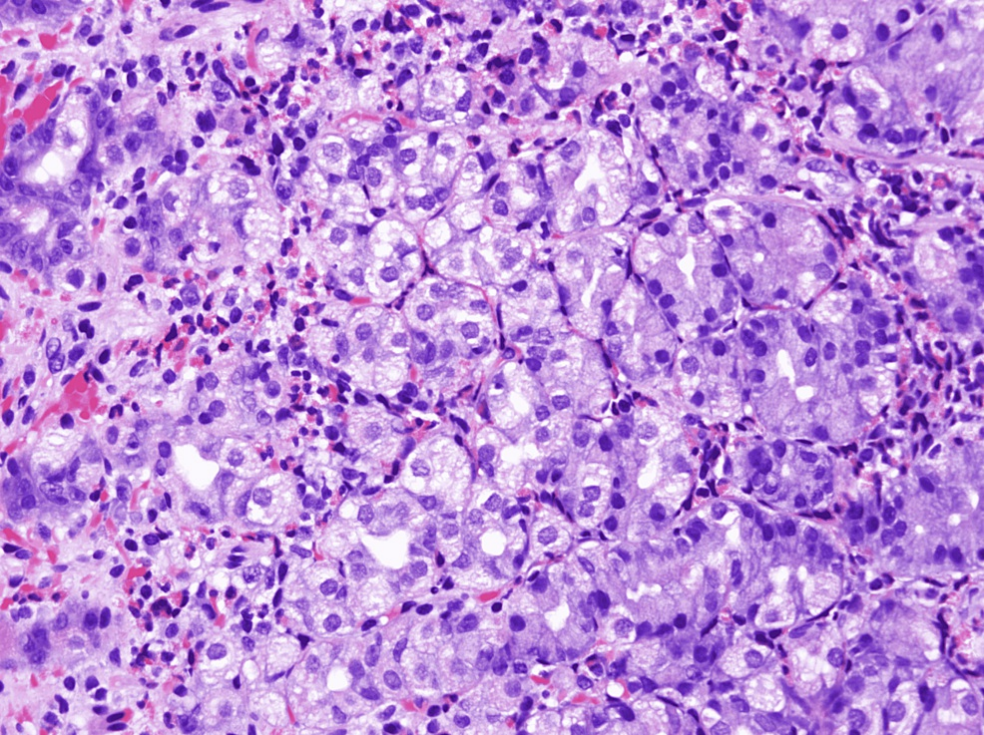
Biopsied gastric body/ulcer sample: high-magnification (400x) view Fragments of gastric body mucosa with moderate chronic gastritis containing numerous eosinophils (>60/high-power field). The eosinophilic infiltrate is arranged in a single layer and small clusters associated with a small focus of superficial erosion.

Biopsies from the gastric body/ulcer revealed chronic inflammation with a predominant eosinophilic infiltrate (up to 60 eosinophils/high-power field) and no intraepithelial lymphocytes (Figures 3, 4). *Helicobacter pylori* stain was negative.

Given the presentation and diagnostic test results, Rheumatology was convinced that the patient had EGPA and recommended initiation of treatment with steroids with the plan for rituximab as an outpatient. The patient was seen in GI and Rheumatology clinics later and had marked improvement in his symptoms and had gained 20 lb in two months. Repeat EGD showed resolution of gross endoscopic abnormalities found on the index procedure and biopsies also confirmed histologic response to the treatment.

## Discussion

Jacob Churg and Lotte Strauss were the first to describe the disease in 1951, which is why EGPA was formerly known as Churg Strauss disease [1]. EGPA is a multisystem disorder characterized by asthma and peripheral eosinophilia. Although EGPA is known to cause small- and medium-vessel vasculitis, it is more common to see just eosinophilic infiltration of the vessel wall rather than necrotizing vasculitis. ANCA is also only positive in only 40-60% of the patients [2,3]. Our patient had negative ANA, p-ANCA, and c-ANCA antibodies but met the clinical criteria for EGPA (asthma, rhino-sinusitis, eosinophils >10%, polyneuropathy). The American College of Rheumatology put forward a few criteria in 1990 for the diagnosis of EGPA. The presence of four or more out of the six features yields a diagnosis of EGPA with a sensitivity of 85% and a specificity of 99.7% [4]:

●Asthma 

●Greater than 10% eosinophils on the differential leukocyte count

●Mononeuropathy (including multiplex) or polyneuropathy

●Migratory or transient pulmonary infiltrates on radiographs 

●Paranasal sinus abnormality

●Biopsy containing a blood vessel showing the accumulation of eosinophils in extravascular areas

EGPA is clinically divided into three different phases although the boundaries are sometimes not distinguishable: (i) prodromal phase with symptoms of atopy, (ii) eosinophilic phase characterized by peripheral eosinophilia and eosinophilic multi-organ involvement, and (iii) vasculitic phase in which small- and medium-vessel vasculitis is seen [5,6]. 

EGPA can invariably involve any organ system but the respiratory tract is almost always involved. The GI tract is involved in 50% of cases of EGPA, with nausea, abdominal pain, and diarrhea being the commonly reported symptoms [7]. The involvement of the GI tract by EGPA can manifest as mucosal erythema, ulceration, perforation, obstruction, and ileus of the GI tract. Of note, GI involvement is considered to be a negative prognostic sign [8].

Although an eosinophilic infiltrate on an organ biopsy can be seen with a cohort of diseases, including EGPA and hypereosinophilic syndrome, our patient satisfied the clinical criteria for EGPA. The characteristic findings of vasculitis are often not seen on biopsy samples potentially because either some cases have not progressed to the vasculitic phase of the disease or the endoscopic biopsy samples are not deep enough to catch the vasculitis. The mainstay of treatment includes steroids and steroid-sparing immunosuppressants. 

## Conclusions

It is interesting to note that the symptoms caused by EGPA-associated inflammation of the GI tract are very nonspecific and can occur with many of the other common inflammatory, infectious, and malignant disorders of the GI tract. Therefore, it is extremely important to keep a broad differential when approaching such a case to diagnose the disease correctly and treat it appropriately.
